# Clinical Features and Risk Factors for Gastrointestinal Complications in Dogs Treated Surgically for Thoracolumbar Intervertebral Disc Extrusion

**DOI:** 10.3389/fvets.2021.785228

**Published:** 2021-12-06

**Authors:** Jaya M. Mehra, M. Katherine Tolbert, George E. Moore, Melissa J. Lewis

**Affiliations:** ^1^Department of Veterinary Clinical Sciences, College of Veterinary Medicine, Purdue University, West Lafayette, IN, United States; ^2^Gastrointestinal Laboratory, Department of Small Animal Clinical Sciences, Texas A&M University, College Station, TX, United States; ^3^Department of Veterinary Administration, College of Veterinary Medicine, Purdue University, West Lafayette, IN, United States

**Keywords:** canine, diarrhea, hemilaminectomy, regurgitation, anti-inflammatories, proton pump inhibitors

## Abstract

Gastrointestinal (GI) complications and their clinical implications are poorly characterized in dogs treated surgically for acute thoracolumbar intervertebral disc extrusion (TL-IVDE). The objective of this retrospective study was to characterize GI signs (including vomiting, diarrhea, melena, and hematochezia) in dogs undergoing hemilaminectomy for acute TL-IVDE. One-hundred and sixteen dogs were included. Frequency, type and severity of GI signs during hospitalization, duration of hospitalization and outcome were obtained from the medical record. Potential risk factors for the development of GI signs were explored using univariable and multivariable analyses. Gastrointestinal signs occurred in 55/116 dogs (47%); 22/55 dogs (40%) had one episode and 21/55 (38%) had ≥5 episodes. Diarrhea was the most common (40/55, 73%) while melena was rare (1/55, 2%). GI signs developed in 8/11 dogs (73%) treated perioperatively with both non-steroidal anti-inflammatories and corticosteroids with or without a washout period and in 25/52 dogs (48%) treated prophylactically with proton pump inhibitors. Median hospitalization was 7 days (4–15 days) vs. 5 days (4–11 days) in dogs with or without GI signs, respectively. Duration of hospitalization was associated with development of any GI signs, diarrhea and more severe GI signs (*p* = 0.001, 0.005, 0.021, respectively). Pre-operative paraplegia with absent pain perception was identified on univariable analysis (*p* = 0.005) and longer anesthetic duration on multivariable analysis to be associated with development of more severe GI signs (*p* = 0.047). In dogs undergoing surgery for acute TL-IVDE, GI signs were common and associated with duration of hospitalization and anesthesia. The influence of specific medications and neurologic severity on development of GI signs requires further investigation.

## Introduction

Intervertebral disc extrusion (IVDE) is the most common cause of acute thoracolumbar (TL) spinal cord injury (SCI) in dogs (1). Gastrointestinal (GI) signs, including diarrhea, vomiting, melena, or hematochezia, are among the reported complications in dogs undergoing surgery for acute thoracolumbar intervertebral disc extrusion (TL-IVDE) (2–5). Colonic perforation (6–8) and pancreatitis (3, 9, 10) have also been reported. Extrapolating from previous studies in dogs treated surgically for acute IVDE, the clinical GI complication rate ranges from 15 to 39% (2–5, 10–12). Similar to dogs, reported complications in people with acute SCI include pancreatitis, GI hemorrhage or ulceration (13–15). Such complications have been associated with prolonged hospitalization, increased cost of care and higher mortality rates (14–16). While much of this is unknown in dogs, mortality attributable to GI signs (specifically hemorrhage) was ~2% in one study (3).

Development of GI complications following SCI is complex and multifactorial. Contributing factors that have been proposed in both people and dogs include more severe neurologic status (2, 3) as well as undergoing hospitalization, anesthesia and surgery (3, 4, 7, 17, 18). Imbalance of the autonomic nervous system (ANS) secondary to disruption of sympathetic outflow (resulting in unchecked parasympathetic output) is also implicated as a cause of GI signs (4, 10, 13, 15–17). In people, this is most commonly associated with cervical and upper thoracic injuries. While not well-characterized in dogs, the preganglionic sympathetic cell bodies are located from T1 to L4/5 suggesting that lesions within the TL region could result in sympathetic outflow dysfunction (3). Additionally, treatment of SCI commonly involves the use of potentially ulcerogenic medications including corticosteroids and non-steroidal anti-inflammatory drugs (NSAIDs) (3–9, 19–21). While GI complications have been reported in dogs with acute TL-IVDE that were treated with high dose corticosteroids (3, 5–7, 9, 12, 22), there is conflicting evidence regarding the association between other anti-inflammatory medications or dosages and the development of GI signs (3, 11, 20, 22–24). Ostensibly to mitigate the perceived risk of development of GI signs in this population, prophylactic treatment with gastro-protectant medications such as proton pump inhibitors (PPIs) has become increasingly widespread; however, evidence to support the benefit of prophylactic PPIs in dogs suffering from acute TL-IVDE is lacking (4, 25).

The primary objectives of this retrospective study were: (1) to describe the frequency, type and severity of GI signs in dogs undergoing hemilaminectomy for acute TL-IVDE and (2) to evaluate potential risk factors for development of GI signs including administration of anti-inflammatory medications, duration of anesthesia or anesthetic complications, duration of hospitalization, severity of initial neurological injury, and prophylactic use of PPIs.

## Materials and Methods

### Case Selection

Medical records from the Purdue University Veterinary Hospital (PUVH) were searched for all dogs that underwent TL hemilaminectomy from May 2017 to January 2020. To be eligible for inclusion, dogs had to have an acute TL-IVDE between T3 and the sacrum diagnosed via computed tomography (CT) or magnetic resonance imaging (MRI) according to published descriptions (26) and confirmed surgically via the presence of extradural, extruded disc material within the spinal canal. Dogs were included if their onset of gait deficits occurred ≤ 14 days prior to presentation, however, dogs with back pain only for ≤ 21 days, with or without subsequent development of gait deficits, were also included. Dogs with incomplete medical records or with preexisting conditions that might predispose them to GI signs were excluded. Examples of such preexisting conditions included hepatic disease, renal disease or primary GI disease (e.g., inflammatory bowel disease). Dogs with a history of pancreatitis prior to the onset of neurological deficits were excluded. However, dogs that developed GI signs, including clinical signs of pancreatitis, at the same time as or shortly after the onset of neurological deficits were included. Dogs enrolled in a concurrent prospective clinical trial investigating the effect of prophylactic omeprazole on the development of GI signs were excluded.

### Clinical Information

Breed, sex, age, and body weight were recorded, with additional information obtained from the medical record as outlined below.

### Neurologic Examination

Neurological status was assigned using the Modified Frankel Scale (MFS), with grade 1 = back pain only, grade 2 = ambulatory paraparesis, grade 3 = non-ambulatory paraparesis, grade 4 = paraplegic with intact pain perception, and grade 5 = paraplegic with absent pain perception. MFS at presentation (pre-operatively) and at the time of discharge were recorded. Duration of neurological abnormalities was defined as the period (in days) between the onset of gait deficits or back pain (if pain only) and presentation to the PUVH.

### Gastrointestinal Signs

Any GI signs documented in the medical record during hospitalization (including treatment sheets, daily progress notes, and client communication logs) were recorded for each dog. GI signs were defined as diarrhea, vomiting, regurgitation, melena, or hematochezia. Appetite and nausea were not included due to their subjective nature or inconsistent recording in the medical record. The type of diet fed during hospitalization was recorded. Any GI signs that occurred prior to presentation and were noted in the referring veterinarian's records or in the patient's history were also recorded. The presence of GI signs (yes/no) and severity, defined by the number of episodes of each type of GI sign that occurred during hospitalization, was tabulated as 1, 2–4, or ≥5 episodes. Any treatments instituted due to the development of GI signs were recorded as indicated below.

### Medications

All medications administered during hospitalization were recorded including medication type, dosage and duration of treatment in days. Anti-inflammatory medications (NSAIDs or corticosteroids) administered during the perioperative period were noted, including if more than one type or class was administered. The perioperative period included administration prior to presentation (at the time of onset of pain or neurologic deficits), during hospitalization (pre- or post-operatively) and after discharge (up to 2 weeks). Duration of anti-inflammatory administration was defined as the total number of days the dog received an anti-inflammatory drug during the perioperative period (even if they were non-consecutive days). Gastrointestinal medications including anti-emetics, antacids, prokinetics, and anti-diarrheal medications were recorded when administered. Proton pump inhibitor use was categorized as prophylactic (initiated before onset or in the absence of the development of GI signs), or therapeutic (instituted after GI signs began). If it was not possible to determine this from the medical records, the reason for PPI use was classified as unknown. All GI medications, including PPIs, that were continued post discharge were recorded. Other medications administered as part of diagnosis and treatment for TL-IVDE were recorded. This included opioids and other analgesics, muscle relaxants, and medications for urinary incontinence.

### Imaging and Surgery

Magnetic resonance imaging or CT under general anesthesia were performed to confirm the diagnosis of TL-IVDE. Thoracolumbar hemilaminectomy was performed in standard fashion by a neurology or surgery resident and supervised by a board-certified veterinary neurologist. The duration (in hours) of general anesthesia and specific anesthetic and perioperative medications were recorded. Intraoperative hypotension (defined as a systolic blood pressure <80 mmHg on at least 2 consecutive readings performed 5 min apart) was recorded, as were any other intraoperative complications.

### Duration of Hospitalization and Short-Term Outcome

Survival to discharge and duration of hospitalization (in days) were recorded. For any dogs that did not survive to discharge, the reason and whether it was thought to be related to the development of GI signs (yes/no) was recorded. If available, development of GI signs and any GI medications administered post-discharge were noted.

### Statistical Analysis

Analyses were performed using STATA SE, v.16.1 (StataCorp, College Station, TX). Patient data and frequency and type of GI signs were presented descriptively. Continuous data were assessed for normality using a Shapiro-Wilk test. Normally distributed numerical data were presented as mean (± SD); all other numerical data were presented as median (range). Logistic regression models were used to evaluate potential risk factors for the development of any GI signs (yes/no), development of diarrhea (yes/no), and severity of GI signs (<5 or ≥5 episodes). Diarrhea was evaluated separately due to the frequency of occurrence and since it is a common sequela to many medications utilized in this population. The following variables were evaluated as potential risk factors in the models: severity of neurologic status (ambulatory vs. non-ambulatory; pain perception present vs. absent), duration (in days) and type of anti-inflammatory use (NSAID vs. steroid vs. both), duration of anesthesia (in hours), presence of intraoperative hypotension (yes/no), prophylactic PPI administration (yes/no), concurrent use of prophylactic PPIs and NSAIDs (NSAIDs only vs. prophylactic PPIs only vs. both concurrently) and duration of hospitalization (in days). Independent variables with *p* < 0.2 on univariable analysis were included in a multivariable logistic regression model. Logistic regression models were assessed for goodness-of-fit by the Hosmer-Lemeshow test. *P* < 0.05 was considered significant.

## Results

### Study Population

One-hundred and fifty-three dogs underwent hemilaminectomy between May 2017 and January 2020. Thirty-seven dogs were excluded; 21 were enrolled in a concurrent prospective clinical trial investigating the effect of prophylactic omeprazole on the development of GI signs, 9 dogs underwent hemilaminectomy for a condition other than acute TL-IVDE, and 7 dogs had concurrent medical conditions predisposing them to GI signs or bleeding (3 with GI foreign bodies, 1 with Von Willebrand's Disease, 1 with a recent anal sacculectomy, 1 with recurrent pancreatitis and 1 with acute kidney injury). The remaining 116 cases were included. Three dogs had 2 separate surgeries (>6 months apart) and were each included twice, as independent cases. The median age was 6 years (2–15 years) with a median body weight of 8.2 kg (3.3–45 kg). The most common breed was Dachshund (42/116, 36%), followed by mixed breed dogs (32/116, 28%) and French Bulldogs (7/116, 6%). Eighteen breeds were represented by ≤ 4 dogs. Seventy-two dogs were male (67 neutered, 5 intact) and 44 were female (41 spayed, 3 intact). The median duration of gait deficits was 1 day (1–14 days). One dog had back pain for 1 day with no subsequent gait deficits. Twenty-two dogs had back pain for 1–20 days preceding the onset of neurological signs. The median MFS prior to surgery was grade 3. One dog was classified as grade 1, 20 were grade 2, 44 were grade 3, 26 dogs were grade 4, and 25 were grade 5.

### Gastrointestinal Signs

Fifty-five out of 116 dogs (47%) developed GI signs during hospitalization with type and frequency of episodes outlined in Table 1. Diarrhea was the most common GI sign, followed by regurgitation. Melena was rare. One dog with multiple episodes of hematochezia became anemic (HCT = 29%) but did not require specific therapy. Twenty-two dogs (40%) had a single episode, 12 (22%) had 2–4 episodes and 21 (38%) had ≥5 episodes of any GI signs. Gastrointestinal complications stratified by pre-surgery MFS are depicted in Table 2. The frequency of more severe GI signs (≥5 episodes) increased as the severity of SCI worsened. Thirty-six dogs (65%) had only 1 type of GI sign, 14 dogs (25%) had 2 types, and 5 dogs (9%) had 3 types. Prior to presentation, 10/116 dogs (9%) had GI signs (vomiting in 4, diarrhea in 6); the onset of these signs was concurrent with or after the onset of neurological deficits in all dogs. Six of these dogs had additional GI signs while hospitalized, and were included in the 55 dogs with GI signs, while four did not. Diets fed during hospitalization were typically canned and included Purina Canine Turkey and Sweet Potato, Canine EN, or canned Canine Chicken and Rice entree, Royal Canin Canine Gastrointestinal Low Fat, Hill's Canine i/d, or plain diced chicken (Gordon Food Service).

**Table 1:** Type & frequency of GI signs in 55 of 116 dogs undergoing surgery for TL-IVDE.

**Number of episodes**	**Number of dogs with each GI sign**				
	**Vomiting**	**Diarrhea**	**Regurgitation**	**Melena**	**Hematochezia**
1 episode	6	17	9	1	10
2–4 episodes	4	8	5	0	1
≥5 episodes	0	15	4	0	0
Total (any episodes)	10/55 (18%)	40/55 (73%)	18/55 (33%)	1/55 (2%)	11/55 (20%)

**Table 2:** Gastrointestinal signs stratified by neurologic injury severity in 116 dogs undergoing surgery for TL-IVDE.

**MFS**	**Any GI signs (%)**	**≥5 episodes (%)**
1	0/1 (0)	0/1 (0)
2	11/20 (55)	2/20 (10)
3	14/44 (32)	3/44 (7)
4	16/26 (62)	7/26 (27)
5	14/25 (56)	9/25 (36)

*MFS: modified frankel score*.

### Treatment With Anti-inflammatory Medications

One hundred and seven out of 116 dogs (92%) were treated with anti-inflammatory medications during the perioperative period. Of these, 74 dogs received only NSAIDs and 22 received only corticosteroids. Eleven dogs were treated with both an NSAID and a corticosteroid. Two of the 11 dogs had an appropriate washout period (>4 days) and 9 lacked a washout between administration of the different medications (≤ 1 day).

Carprofen was administered most frequently (*n* = 55), then meloxicam (*n* = 16); other NSAIDs administered in ≤ 2 dogs included deracoxib, firocoxib, grapiprant, ibuprofen, or aspirin. Four dogs were treated with two different NSAIDs during the perioperative period. The median duration of perioperative NSAID use was 8 days (1–29 days). The most frequently used corticosteroid was oral prednisone, administered by itself in 16 dogs or in combination with an injectable corticosteroid in 9 dogs. Injectable medications included dexamethasone SP, dexamethasone, triamcinolone acetonide, cortisone, and methylprednisolone sodium succinate. Corticosteroid doses varied widely and could not be verified in all cases. The median duration of perioperative corticosteroid treatment was 2 days (1–23 days).

Table 3 outlines GI signs during hospitalization in dogs treated with anti-inflammatory medications. Of the 4 dogs that received two different NSAIDs during the perioperative period, one dog had a single episode of regurgitation.

**Table 3:** Frequency of GI signs in 107 of 116 dogs undergoing surgery for TL-IVDE and receiving perioperative anti-inflammatory medications.

**Anti-inflammatory administered**	**Any GI signs**	**Vomiting**	**Diarrhea**	**Regurgitation**	**Melena**	**Hematochezia**
NSAID (*n* = 74)	34 (46%)	7	21	13	1	6
Carprofen (*n* = 55^*^)	27 (49%)	5	18	12	1	3
Meloxicam (*n* = 16^*^)	6 (38%)	1	3	1	0	2
Steroid (*n* = 22)	9 (41%)	0	8	1	0	3
Both NSAID and steroid (*n* = 11)	8 (73%)	2	7	1	0	0

* *Four dogs in the carprofen group and one dog in the meloxicam group were treated with >1 NSAID; one dog (treated with carprofen and ibuprofen) had a single episode of regurgitation*.

### Treatment of Gastrointestinal Signs

Eight out of 116 dogs (7%) were treated with GI medications prior to presentation including maropitant (*n* = 4), famotidine (*n* = 7), sucralfate (*n* = 1), ranitidine (*n* = 1), capromorelin (*n* = 1), bismuth subsalicylate (*n* = 1) and metronidazole (*n* = 1). Three dogs received these medications to treat overt GI signs while 5 dogs were treated for non-specific signs (e.g., pain, tense abdomen, hunched back) in the absence of overt GI signs. No dogs received PPIs prior to presentation. Four of 8 dogs that received GI medications prior to presentation were administered a PPI at our institution; the reason for PPI administration was classified as unknown.

One hundred and eight dogs did not receive any GI medications prior to presentation. Of these, 64 dogs (59%) were administered PPIs during hospitalization and 44 dogs (41%) were not. Proton pump inhibitors were administered prophylactically in 52/64 dogs (81%) and were administered therapeutically in 12/64 dogs (19%). Maropitant (1 mg/kg SQ) was administered as a routine pre-anesthetic medication in 48/116 dogs (41%), including 24/52 dogs (46%) that received prophylactic PPIs and 16/44 dogs (36%) that did not receive a PPI. The use of pre-anesthetic maropitant was not significantly different between dogs receiving prophylactic PPIs and those that did not receive PPIs (*p* = 0.4). In the 52 dogs receiving prophylactic PPIs, treatment protocols varied but generally consisted of ~1 mg/kg q12–24 h of pantoprazole (*n* = 28), pantoprazole then omeprazole (*n* = 23) or omeprazole (*n* = 1) for varying lengths of time (1–12 days). Table 4 outlines the frequency of GI signs in the dogs receiving prophylactic PPIs compared to those not administered a PPI. There was no difference in the frequency of GI signs between dogs treated with PPIs once or twice-daily (*p* = 0.55).

**Table 4:** Frequency of GI signs in 96 of 116 dogs undergoing surgery for TL-IVDE with or without prophylactic PPI administration.

**PPI administration**	**Any GI signs**	**Vomiting**	**Diarrhea**	**Regurgitation**	**Melena**	**Hematochezia**
Prophylactic PPI (*n* = 52)	25 (48%)	5 (10%)	20 (38%)	8 (15%)	0	5 (10%)
No PPI (*n* = 44)	16 (36%)	1 (2%)	12 (27%)	4 (9%)	0	3 (7%)

Thirty-four of 55 dogs (62%) received specific medical therapy for their GI signs during hospitalization. Doses and duration varied but medications administered included PPIs (*n* = 12), maropitant (*n* = 27), metronidazole (*n* = 19), metoclopramide (*n* = 8), famotidine (*n* = 2), ondansetron (*n* = 3), sucralfate (*n* = 1), or capromorelin (*n* = 1). Sixteen dogs (29%) with GI signs received no specific therapy. Five additional dogs were treated with GI medications (other than prophylactic PPIs) during hospitalization despite no GI signs documented in the medical record; the reason for administration was unclear.

### General Anesthesia, Surgery, and Post-operative Care

Anesthetic protocols varied between dogs; pre- and intra-operative medications included injectable opioids (fentanyl, hydromorphone, methadone) with intermittent use of ketamine, lidocaine and anticholinergic medications. Additional perioperative medications included gabapentin, acepromazine, dexmedetomidine, trazodone, methocarbamol, prazosin, or diazepam in variable numbers of dogs. The mean duration of anesthesia was 4.8 h (+/−1.2), and the median duration of surgery was 2.6 h (1.3–5.6 h).

Twenty-eight out of 116 dogs (24%) had at least one 5 min period of hypotension under general anesthesia. In 7 dogs, the hypotension was limited to a single 5 min period and 21 dogs had one or more periods of hypotension cumulatively lasting ≥10 min (range: 10–60 min); in 21 dogs, the systolic blood pressure was 60–80 mmHg during the period of hypotension and in 7 dogs at least one reading of <60 mmHg was obtained during the hypotensive period. GI signs occurred in 11/28 dogs (39%) with hypotension compared to 44/88 dogs (50%) without hypotension. Sixteen dogs with documented hypotension received specific treatment consisting of colloid administration (*n* = 4), glycopyrrolate (*n* = 6), atropine (*n* = 8), or a dobutamine CRI (*n* = 1). Four additional dogs had a variable period of time during anesthesia where an indirect blood pressure could not be measured, due to patient or equipment-related issues.

Two dogs were suspected to develop aspiration pneumonia post-operatively (during hospitalization), both of which had at least 1 episode of vomiting post-operatively. Both dogs received pantoprazole during hospitalization with its use classified as treatment in 1 and unknown in the other.

Post-operative care in all dogs included activity restriction, analgesia, and basic physical rehabilitation during hospitalization. Bladder management was performed as needed and consisted of manual expression or placement of an intermittent or indwelling urinary catheter.

### Outcome and Follow-Up

One hundred and ten dogs survived to discharge and 6 were euthanized during hospitalization. Five dogs were euthanized due to suspected progressive myelomalacia; 2 had GI signs and 3 did not. One dog developed aspiration pneumonia and hemorrhagic diarrhea and was euthanized 3 days post-operatively due to suspected sepsis. Post-mortem examination was not performed and the cause of sepsis (respiratory or GI) could not be confirmed. The median duration of hospitalization for all surviving dogs was 6 days (4–15 days). Among dogs with GI signs, the median duration of hospitalization was 7 days (4–15 days) compared to 5 days (4–11 days) for dogs without GI signs. Forty-two of 55 dogs (76%) that developed GI signs were hospitalized for ≥6 days.

Seventeen dogs were discharged with GI medications including 11 dogs treated prophylactically with PPIs during hospitalization. Two of 11 dogs had no GI signs in the hospital and were both discharged with omeprazole. Nine of 11 dogs developed variable GI signs (3/9 vomiting, 8/9 diarrhea, 5/9 regurgitation, 1/9 hematochezia) during hospitalization and were discharged with omeprazole (*n* = 5) and metronidazole (*n* = 4). The 6 remaining dogs had GI signs (1/6 vomiting, 4/6 diarrhea, 2/6 regurgitation, 1/6 hematochezia) in the hospital and were discharged with metronidazole (*n* = 3), omeprazole (*n* = 2), and maropitant (*n* = 1).

Follow-up information was available in 90 dogs. Two dogs were euthanized within 1 week post-operatively, one due to suspected progressive myelomalacia and the other at the owner's wishes due to failure to regain pain perception. GI complications were not the primary reason for euthanasia in either dog. GI signs requiring re-hospitalization or prolonged therapy (>10 days) were not noted for any dog.

### Risk Factor Assessment

Tables 5–7 outline logistic regression analyses of the previously stated potential risk factors for the development of any GI signs, the development of diarrhea, and the severity of GI signs. Longer duration of hospitalization was associated with an increased frequency of GI signs, increased frequency of diarrhea and increased frequency of more severe GI signs (*p* = 0.001, 0.005, 0.021, respectively). Additionally, longer duration of anesthesia was associated with the development of more severe GI signs (*p* = 0.047). On univariable analysis, pre-operative paraplegia with absent pain perception was associated with the development of more severe GI signs (*p* = 0.005) but this was no longer significant on multivariable analysis (*p* = 0.374). No other significant relationships were identified including medications or combinations of medications administered.

**Table 5:** Univariable and multivariable analysis of risks factors for the development of GI signs in 116 dogs undergoing surgery for TL-IVDE.

**Variable**	***N***	**GI signs**	**GI signs**	**Univariable**		**Multivariable**	
		**Yes (%)**	**No (%)**				
				**OR (95% CI)**	***p*-value**	**OR (95% CI)**	***p*-value**
**Neurological severity**							
Non-ambulatory	116	44 (46.3)	51 (53.7)	0.78 (0.30–2.02)	0.615		
Ambulatory		11 (52.4)	10 (47.6)				
Pain perception absent	116	14 (60.9)	9 (39.1)	1.97 (0.78–5.01)	0.153	1.47 (0.37–5.81)	0.583
Pain perception present		41 (44.1)	52 (55.9)				
**Anesthesia**							
Duration (hours)	112^#^	NA	NA	1.26 (0.90–1.75)	0.172	1.24 (0.70–2.19)	0.179
Intraoperative hypotension yes	116	11 (39.3)	17 (60.7)	0.65 (0.27–1.54)	0.324		
Intraoperative hypotension no		44 (50.0)	44 (50.0)				
**Anti-inflammatory use**							
Duration (days)	116	NA	NA	1.02 (0.97–1.08)	0.381		
None	116	4 (44.4)	5 (55.6)				
Corticosteroid		9 (40.9)	13 (59.1)	1	NA	1	NA
NSAID		34 (46.0)	40 (54.0)	1.23 (0.47–3.22)	0.677	3.86 (0.37–40.0)	0.258
Both		8 (72.7)	3 (27.3)	3.85 (0.80–18.62)	0.093	9.36 (0.36–244.8)	0.179
**PPIs**							
Prophylactic PPI	96^∧^	25 (48.1)	27 (51.9)	1.85 (0.82–4.16)	0.136	1.54 (0.45–5.30)	0.489
No PPI		16 (33.3)	28 (66.7)				
No PPI or NSAID	92^**^	4 (36.4)	7 (63.6)				
NSAID only		11 (30.6)	25 (69.4)	1	NA	1	NA
PPI only		7 (41.2)	10 (58.8)	1.59 (0.48–5.27)	0.448	1 (collinearity)	NA
Both		13 (46.4)	15 (53.6)	1.97 (0.71–5.50)	0.196	1 (collinearity)	NA
**Hospitalization**							
Duration (days)	116	NA	NA	1.53 (1.23–1.90)	<0.001^*^	1.85 (1.31–2.63)	0.001^*^

# *N = 112 excludes dogs where imaging and surgery occurred under separate anesthetic episodes*.

∧ *N = 96 excludes dogs in which PPI treatment was therapeutic or in which reason for administration was unknown*.

** *N = 92 excludes dogs treated with both NSAIDs and corticosteroids or where PPI administration was therapeutic or unknown*.

* *p < 0.05*.

**Table 6:** Univariable and multivariable analysis of risk factors for the development of diarrhea in 116 dogs undergoing surgery for TL-IVDE.

**Variable**	***N***	**Diarrhea**	**Diarrhea**	**Univariable**		**Multivariable**	
		**yes (%)**	**no (%)**				
				**OR (95% CI)**	***p*-value**	**OR (95% CI)**	***p*-value**
**Neurological severity**							
Non-ambulatory	116	33 (34.7)	62 (65.3)	1.06 (0.39–2.90)	0.903		
Ambulatory		7 (33.3)	14 (66.7)				
Pain perception absent	116	10 (43.5)	13 (56.5)	1.62 (0.64–4.10)	0.313		
Pain perception present		30 (32.3)	63 (67.7)				
**Anesthesia**							
Duration (hours)	112^#^	NA	NA	1.22 (0.87–1.72)	0.244		
Intraoperative hypotension yes	116	9 (32.1)	19 (67.9)	0.87 (0.35–2.15)	0.765		
Intraoperative hypotension no		31 (35.2)	57 (64.8)				
**Anti-inflammatory use**							
Duration (days)	116	NA	NA	1.02 (0.97–1.07)	0.512		
None	116	4 (44.4)	5 (55.6)				
Corticosteroid		8 (36.4)	14 (63.6)	1	NA	1	NA
NSAID		21 (28.4)	53 (71.6)	0.69 (0.25–1.89)	0.475	0.86 (0.18–4.09)	0.853
Both		7 (63.6)	4 (36.4)	3.06 (0.68–13.79)	0.145	4.37 (0.36–52.8)	0.246
**PPIs**							
Prophylactic PPI	96^∧^	20 (38.5)	32 (61.5)	1.67 (0.70–3.97)	0.248		
No PPI		12 (25.0)	32 (75.0)				
No PPI or NSAID	92^**^	3 (27.3)	8 (72.7)				
NSAID only		8 (22.2)	28 (77.8)	1	NA	1	NA
PPI only		7 (41.2)	10 (58.8)	2.45 (0.71–8.51)	0.158	1 (collinearity)	NA
Both		9 (32.1)	19 (67.9)	1.66 (0.54–5.06)	0.375	1.23 (0.37–4.07)	0.735
**Hospitalization**							
Duration (days)	116	NA	NA	1.27 (1.07–1.50)	0.006^*^	1.40 (1.10–1.78)	0.005^*^

# *N = 112 excludes dogs where imaging and surgery occurred under separate anesthetic episodes*.

∧ *N = 96 excludes dogs in which PPI treatment was therapeutic or in which reason for administration was unknown*.

** *N = 92 excludes dogs treated with both NSAIDs and corticosteroids or where PPI administration was therapeutic or unknown*.

* *p < 0.05*.

**Table 7:** Univariable and multivariable analysis of risk factors for the severity of GI signs (≥ or <5 episodes) in 116 dogs undergoing surgery for TL-IVDE.

**Variable**	***N***	**Frequency ≥5 (%)**	**Frequency <5 (%)**	**Univariable**		**Multivariable**	
				**OR (95% CI)**	***p*-value**	**OR (95% CI)**	***p*-value**
**Neurological severity**							
Non-ambulatory	116	19 (20.0)	76 (80.0)	2.38 (0.51–11.09)	0.271		
Ambulatory		2 (9.5)	19 (90.5)				
Pain perception absent	116	9 (39.1)	14 (60.9)	4.34 (1.54–12.20)	0.005^*^	2.56 (0.32–20.26)	0.374
Pain perception present		12 (12.9)	81 (87.1)				
**Anesthesia**							
Duration (hours)	112^#^	NA	NA	1.62 (1.06–2.48)	0.025^*^	3.48 (1.01–11.95)	0.047^*^
Intraoperative hypotension yes	116	2 (7.1)	26 (92.9)	0.28 (0.06–1.28)	0.101	1 (collinearity)	NA
Intraoperative hypotension no		19 (21.6)	69 (78.4)				
**Anti-inflammatory use**							
Duration (days)	116	NA	NA	0.95 (0.88–1.02)	0.176	0.78 (0.53–1.14)	0.201
None	116	2 (22.2)	7 (77.8)				
Corticosteroid		5 (22.7)	17 (77.3)	1	NA		
NSAID		12 (16.2)	62 (83.8)	0.66 (0.20–2.13)	0.484		
Both		2 (18.2)	9 (81.8)	0.76 (0.12–4.70)	0.764		
**PPIs**							
Prophylactic PPI	96^∧^	10 (19.2)	42 (80.8)	1.86 (0.58–5.92)	0.295		
No PPI		5 (10.4)	39 (89.6)				
No PPI or NSAID	92^**^	2 (18.2)	9 (81.8)				
NSAID only		3 (8.3)	33 (91.7)	1	NA	1	NA
PPI only		4 (23.5)	13 (76.5)	3.39 (0.66–17.33)	0.142	0.36 (0.01–11.20)	0.560
Both		4 (14.3)	24 (85.7)	1.83 (0.38–8.96)	0.454	0.46 (0.04–5.70)	0.542
**Hospitalization**							
Duration (days)	116	NA	NA	1.49 (1.22–1.82)	<0.001^*^	1.83 (1.09–3.06)	0.021^*^

# *N = 112 excludes dogs where imaging and surgery occurred under separate anesthetic episodes*.

∧ *N = 96 excludes dogs in which PPI treatment was therapeutic or in which reason for administration was unknown*.

** *N = 92 excludes dogs treated with both NSAIDs and corticosteroids or where PPI administration was therapeutic or unknown*.

* *p < 0.05*.

## Discussion

Gastrointestinal signs were common in dogs treated surgically for acute TL-IVDE. Almost half of the dogs in this study developed GI signs during hospitalization, with diarrhea being the most common sign. Gross evidence of GI bleeding was uncommon, with only a single case with anemia secondary to hematochezia. The presence, type and severity of GI signs were associated with duration of hospitalization and the severity of GI signs was also associated with anesthetic duration.

The clinical GI complication rate in this study (47%) is higher than previous reports, from which a GI complication rate in dogs with IVDE of 15–39% can be extrapolated (2–5, 10–12). Among prior studies, only a subset focused on reporting clinical GI signs as a main objective (2, 3, 11), and specific details such as frequency, severity or duration of GI signs were often lacking (2, 4, 5, 10, 11). We purposely included dogs with even a single episode of GI signs during hospitalization to broadly characterize the nature and severity of these signs. While our results support that GI signs are common, they were frequently mild, consisting of an isolated episode in 22/55 dogs (40%) and 16/55 dogs (29%) in which no specific therapy was instituted. If only dogs with ≥2 episodes of GI signs are considered, the overall GI complication rate is 28% and decreases to 18% if only dogs with ≥5 episodes are included.

Ninety-five percent of dogs in this study survived to discharge; GI signs could have been the cause or a contributing factor to suspected sepsis and the decision to euthanize in one dog with hemorrhagic diarrhea and aspiration pneumonia. This suggests a possible mortality rate of <1% secondary to GI signs, specifically GI hemorrhage. This is similar to previously reported mortality rates due to GI complications of 2% in dogs with IVDE (3) and 4–15% reported in people with SCI (27–29). Assessing mortality in this population is complicated by euthanasia, difficulty in definitively determining the role of GI signs, and various potential confounding factors (e.g., medications administered); however rare, life-threatening GI complications are possible.

Clinical evidence of GI bleeding was uncommon. Hematochezia was noted in <10% of dogs, the vast majority of which had a single episode, and 1 dog had a single episode of melena. Hematochezia and melena are difficult to quantify from prior studies, but it has been reported that 15% of dogs with IVDE develop GI hemorrhage (3). This finding is confounded by dexamethasone administration which has been implicated in the development of GI hemorrhage (3) and diarrhea (11). Occult evidence of GI bleeding is reported in 90% of dogs undergoing spinal surgery (5). Additionally, endoscopic and histopathologic evidence of GI mucosal injury (ranging from submucosal hemorrhage to overt ulceration) has been demonstrated in 76% of dogs undergoing surgery for TL-IVDE (4, 23). Despite the high prevalence of mucosal injury and occult bleeding, concurrent GI clinical signs including gross evidence of hemorrhage are reported in a much smaller subset of cases or not at all (4, 5, 23). These studies highlight the fact that subclinical GI mucosal lesions are common in this population, but overt hemorrhage is not.

The pathophysiology of the development of GI signs in dogs with acute TL-IVDE is complex and likely multifactorial. While not completely understood, factors that have been proposed include ANS dysfunction (4, 10, 13, 15–17), perioperative stress such as that associated with surgery, hospitalization, and general anesthesia (3, 4, 7, 17, 18), and the use of potentially ulcerogenic medications such as NSAIDs and/or corticosteroids (3–9, 19–21). In our study, longer hospitalization was associated with the development of any GI signs, the development of diarrhea, and the presence of more severe signs. This is consistent with SCI in people in which significantly longer hospitalization is reported in those who have GI complications (14). While causality cannot be determined, it is possible that dogs were hospitalized for longer because of the development of GI signs and the related treatment or subsequent complications. For example, a dog with multiple episodes of vomiting developed pneumonia and was hospitalized for 6 days. Post-anesthetic regurgitation or vomiting are known risk factors for pneumonia (30, 31), the treatment of which could require longer hospitalization. Similarly, clinicians might have been reluctant to discharge dogs that developed diarrhea or such decisions might have been owner-driven leading to more prolonged hospitalization. However, it also remains possible that longer hospitalization itself resulted in more GI signs. A hospital environment and longer duration in the hospital are associated with increased stress in dogs as measured by higher serum cortisol levels (32, 33). Physiologic stress is known to cause stress-related mucosal disease in ill people (16, 34) and has been implicated as a factor in the development of GI lesions and hemorrhage in dogs hospitalized in a veterinary ICU as well as racing Alaskan sled dogs (35, 36). Among the dogs of our study hospitalized for TL-IVDE, those with more prolonged hospitalization might have had increased physiologic stress resulting in breakdown of the GI mucosal barrier and more frequent and severe GI signs.

Severe SCI can disrupt the sympathetic outflow from the central nervous system, resulting in ANS imbalance (unchecked parasympathetic output) that has been implicated in the development of hemorrhage, necrosis, and ulceration of the GI tract (17). In people with SCI, this ANS imbalance is more commonly associated with severe cervical and upper thoracic lesions (14, 15, 17), though this information is not well-characterized in dogs. Since the sympathetic preganglionic fibers in dogs are located extensively from T1 to L4/5, IVDE within the TL region could result in sympathetic outflow dysfunction (3). Autonomic nervous system dysfunction secondary to acute SCI has also been proposed to contribute to the development of pancreatitis, which can cause GI signs (3, 10, 13). Additionally, more severe TL SCI has been associated with a greater likelihood of GI complications suspected, in part, to be due to more pronounced disruption of the ANS (2, 3, 17). Our results provide potential support for this, since the frequency of more severe GI signs increased as the severity of neurologic injury increased. The absence of pain perception pre-operatively was associated with the development of more severe GI signs (≥5 episodes) on univariable analysis. While this association was no longer significant on multivariable analysis, this potential relationship between dogs with more severe initial neurological status having more severe GI signs might support a role for ANS dysfunction in the development of GI signs in dogs with severe SCI. However, it is possible that autonomic imbalance plays a relatively minor role compared to other factors such as stress, surgery, hospitalization, and medications.

Proton pump inhibitors were commonly administered prophylactically, ostensibly to mitigate the risk of GI complications. Prophylactic PPI use has been advocated in people with acute SCI to reduce the risk of developing stress ulcers and GI hemorrhage (34, 37, 38). While their role has not been established in dogs with SCI, previous reports of a high rate of mucosal injury in dogs undergoing surgery for TL-IVDE or receiving anti-inflammatory medications (4, 39) have led to widespread use of PPIs (40). However, prior studies of omeprazole orally administered at 0.7 mg/kg once-daily did not identify a significant reduction in the frequency or progression of such lesions (4, 41) and the relationship between mucosal injury and clinical GI signs remains unclear (4, 23). In our study, PPI administration was not standardized, and the majority of dogs received either a dose lower than the current treatment guidelines of 1 mg/kg PO q12 h or were treated for <2–4 days required to achieve maximal gastric acid suppression (25). Dosing and duration inconsistencies between dogs make it challenging to evaluate the influence of PPIs on the prevention or development of GI signs, but prophylactic PPI administration was not associated with a reduced risk of clinical GI signs. In fact, more dogs receiving PPI prophylaxis developed GI signs, especially diarrhea, compared to dogs that did not receive PPIs. This is consistent with a report in dogs hospitalized in the ICU in which prophylactic administration of gastro-protectants was associated with a significantly increased risk of development of hemorrhagic GI disease (35). The most common adverse effect associated with PPI administration in dogs is diarrhea (42–44), but other more serious complications including intestinal dysbiosis and subsequent bacterial pneumonia are possible (25, 45).

It was not possible to investigate the overall impact of anti-inflammatory medications in this study given the small number of untreated dogs. NSAIDs negatively impact the GI tract primarily by inhibiting prostaglandins, resulting in decreased mucosal resistance to injury (46). Furthermore, NSAIDs that undergo enterohepatic recirculation (e.g., carprofen) might be more likely to cause GI mucosal injury (46), and NSAIDs have been associated with the development of diarrhea (21, 47). Corticosteroids, especially dexamethasone and administration at higher doses, have similarly been implicated in the development of mucosal injury and GI signs in dogs (5, 11, 12, 19, 22, 48, 49), though many dogs in prior studies were also treated with NSAIDs (3–7, 9, 12, 23). The combination of NSAIDs and steroids has been shown to increase GI mucosal injury (50) which might explain the higher frequency of GI complications in dogs of this study treated with both NSAIDs and steroids. However, this was not identified as a risk factor, perhaps attributable to the study being underpowered to investigate this, the lack of standardization regarding dose and duration of NSAID or corticosteroid therapy and other potential confounding factors such as the impact of anesthetic duration, hospitalization, or other medications administered.

There is also growing evidence in people and dogs that concurrent administration of PPIs and NSAIDs can increase the risk of GI injury, permeability and inflammation, though this does not necessarily translate to clinical GI signs in all cases (45, 51, 52). While our results demonstrated that more dogs receiving both NSAIDs and prophylactic PPIs developed GI signs (46%) compared to either individually (41 and 30%), this difference was not significant. This might be due to small numbers of dogs in each group, other confounding variables (e.g., anesthetic or hospitalization duration) and the fairly high percentage of GI signs in dogs treated with prophylactic PPIs alone (41%). While none of the dogs treated with NSAIDs, prophylactic PPIs or combinations of those medications suffered life-threatening GI complications, the possibility and specific circumstances under which anti-inflammatory medications or PPIs might contribute to the development of GI signs in dogs with acute TL-IVDE remain to be more clearly elucidated.

We did not identify an association between anesthetic duration and the overall development of GI signs, but longer duration of anesthesia was associated with increased severity of GI signs. The etiology of this finding is likely multifactorial. Longer duration of general anesthesia has been associated with gastroesophageal reflux, regurgitation and post-operative vomiting (24). Opioids, especially pure μ agonists, administered perioperatively can result in ileus (53) and increase the likelihood of gastroesophageal reflux during anesthesia (54, 55). While anesthetic protocols varied, opioids were utilized in the vast majority of patients, potentially increasing the risk of regurgitation. Intra-operative hypotension has also been postulated to contribute to the development of GI signs (19, 22). In our study, hypotension was not associated with an increased risk or severity of GI signs. However, only 7 dogs experienced severe hypotension and most dogs had non-invasive (indirect) blood pressure monitoring. Furthermore, there were occasional periods of time where blood pressure measurements were not collected due to factors such as small patient size or equipment malfunction. These limitations and inconsistencies in monitoring might have impacted the ability to assess the role of hypotension under general anesthesia on the development of GI signs.

The main limitation of this study is its retrospective nature. It is possible that some GI signs were not captured in the medical record or that episodes were misclassified. Occasionally, GI signs were not described in extensive detail, limiting interpretation of severity, or clinical significance. It was also difficult to evaluate when or how therapy was initiated for the prevention or treatment of GI signs. We deemed that PPIs were prophylactically administered if the first dose was administered before the onset or in the absence of any documented GI signs, but it is possible that some dogs were misclassified. Similarly, the decision to institute treatment for GI signs was clinician dependent, resulting in variable severity of GI signs prior to initiation of treatment and variable medication regimens. In general, the lack of standardized medication protocols (relating to treatment associated with TL-IVDE or treatment or prevention of GI signs) might have influenced the development and progression of GI signs among dogs and is a notable limitation. Given the large point estimates of the odds ratios associated with many of the medications (or combinations of medications) included in our logistic regression analysis, underpowered subgroups are a likely factor. Additionally, large changes in odds ratios between univariable and multivariable analyses of the various medications are indicative of confounding. As such, our results should be interpreted with caution and signify that prospective studies are needed, in which various potential confounding factors can be controlled, to discern the impact of one or more of these medications on the development of GI signs in dogs with TL-IVDE. While not an objective of this study, we were also not able to determine if there are unique factors predisposing to GI signs in dogs with surgically-managed TL-IVDE or if such signs are simply attributable to general features of surgery, anesthesia and hospitalization that could be encountered in any dog undergoing surgery for a painful, non-GI-related condition. However, the high rate of endoscopically identified mucosal injury and frequent prophylactic treatment for potential GI signs in dogs with TL-IVDE managed surgically justifies evaluation of this study population specifically.

In a group of dogs undergoing hemilaminectomy for acute TL-IVDE, duration of hospitalization and anesthetic duration were associated with the development of GI signs, but serious or life-threatening complications were rare. The impact of initial severity of neurologic status and the role of certain medications including anti-inflammatories and the prophylactic use of PPIs requires further evaluation.

## Data Availability Statement

The raw data supporting the conclusions of this article will be made available by the authors, without undue reservation.

## Ethics Statement

Ethical review and approval was not required for the animal study because it was a retrospective study. Written informed consent for participation was not obtained from the owners because it was a retrospective study.

## Author Contributions

JM, MT, and ML participated in generation of study concept and experimental design, data collection and analysis, manuscript preparation, and editing. GM participated in data analysis, manuscript preparation, and editing. All authors contributed to the article and approved the submitted version.

## Conflict of Interest

The authors declare that the research was conducted in the absence of any commercial or financial relationships that could be construed as a potential conflict of interest.

## Publisher's Note

All claims expressed in this article are solely those of the authors and do not necessarily represent those of their affiliated organizations, or those of the publisher, the editors and the reviewers. Any product that may be evaluated in this article, or claim that may be made by its manufacturer, is not guaranteed or endorsed by the publisher.
